# The correlation between multiple HPV infections and the occurrence, development, and prognosis of cervical cancer

**DOI:** 10.3389/fmicb.2023.1220522

**Published:** 2023-07-28

**Authors:** Jing Na, Ya Li, Jun Wang, Xinyou Wang, JunLing Lu, Shichao Han

**Affiliations:** Department of Gynecology and Obstetrics, Second Affiliated Hospital of Dalian Medical University, Dalian, China

**Keywords:** multiple HPV infections, cervical cancer, occurrence, development, prognosis

## Abstract

Cervical carcinoma is the fourth female malignant tumor in the world, and the persistent infection of high-risk human papillomavirus (HPV) is recognized as the most common cause. This article studies the correlation between multiple HPV infections and the occurrence, development, and prognosis of cervical cancer in order to provide more references for clinical diagnosis and treatment. We conducted a retrospective analysis of the clinical data of 400 cervical carcinoma patients admitted to our hospital from 2015 to 2023. The collected patient data include age, HPV infection status, tumor size and morphology, local infiltration depth, diagnostic staging, surgical approach, vascular cancer thrombus status, lymph node status, and postoperative HPV follow-up status. We use SPSS statistical software for data analysis. Our research shows that the high-risk age group for cervical carcinoma is concentrated between 41 and 60 years old, which is basically consistent with the age range of the high incidence of HPV infection. In the statistics for HPV infection types, ~67.7% of patients are single HPV-infected, 25.29% are double infected, and 7.00% are infected with three or more types of HPV. Among the multiple HPV infections, most of the patients are younger than 40 years old and older than 70 years old, with double infection accounting for the majority. The top five HPV subtypes with high detection rates belong to high-risk subtypes, which are the HPV16, 18, 58, 33, and 52 subtypes, respectively. There was no significant relationship between multiple HPV infections and cervical cancer stage, lesion size, pathological tissue type, tissue differentiation degree/vascular cancer thrombus, and lymph node metastasis, and there was no significant difference in the results between the groups. In summary, multiple types of HPV infection in the cervix are common. We found that multiple infections, mainly HPV16, are closely related to cervical cancer. For the HPV16, 18, 58, 33, and 52 subtypes of infection, especially for patients younger than 40 years old and older than 70 years old, priority should be given to prevention and treatment. The relationship between multiple HPV infections and the progression and prognosis of cervical carcinoma requires further research, which could better guide cancer prevention and treatment.

## Introduction

Cervical carcinoma is the fourth female malignant tumor in the world, and it is also the most usual malignant tumor of the female reproductive tract. Its morbidity is only next to breast cancer, colorectal cancer, and lung cancer (Hu and Ma, 2018; Arbyn et al., 2020). At present, the morbidity of cervical carcinoma in developing countries is still rising year by year. The occurrence of cervical carcinoma usually goes through a long process, which is the result of multiple factors, multiple genes, and multi-step long-term joint action. The human papillomavirus (HPV) is closely related to the occurrence of cervical carcinoma, and the persistent infection of high-risk HPV is currently recognized as the most common reason for cervical carcinoma (de Freitas et al., 2015). Most women infected with HPV can be cleared by their own immune function, and only a small portion of HPV infection may cause cervical lesions or even cervical cancer (Akeel, 2015; Husain and Ramakrishnan, 2015). Due to the different pathogenic potential of different subtypes of HPV infection (Salazar et al., 2015), the risk of cervical carcinoma and cervical lesions varies. Multiple infections refer to the simultaneous infection of two or more HPV subtypes. Meanwhile, due to the lack of cross antibodies between different subtypes of HPV, multiple HPV infections are common. Therefore, the correlation between multiple HPV infections and cervical carcinoma and cervical lesions is receiving increasing attention. There is currently no consensus on whether multiple HPV infections will increase the incidence of cervical carcinoma, and there is no consensus on their impact on cervical carcinoma. This article studies the correlation between multiple HPV infections and the occurrence, development, and prognosis of cervical cancer in order to provide more reference for clinical diagnosis and treatment.

## Methods

A retrospective analysis was conducted on the clinical data of 400 cervical carcinoma patients admitted to our hospital from 2015 to 2023. The inclusion criteria for cases are pathological diagnoses of cervical cancer, while the exclusion criteria are immune system diseases, chronic wasting diseases, and organ transplant surgery. The collected patient data include age, menopausal status, HPV infection status, tumor size and morphology, local infiltration depth, diagnostic staging, surgical approach, postoperative pathological type, interstitial infiltration depth, vascular cancer thrombus status, lymph node status, lymphatic space involvement (LVSI), squamous cell carcinoma antigen (SCC), smoking history, postoperative adjuvant treatment, postoperative HPV follow-up status, tumor-free survival rate, follow-up interval, and mortality rate. All information is obtained by consulting medical records. This study was approved by the Ethics Committee of the Second Affiliated Hospital of Dalian Medical University. This study used SPSS software version 25.0 for statistical analysis. Heterogeneity comparison between groups was conducted using the chi-square test, and Fisher's exact test was used when a single sample size was < 4. For the remaining analyses, a *p*-value of < 0.05 was considered statistically significant.

## Results

### Analysis of clinical data characteristics of cervical cancer patients

We described the clinical data of 400 cervical carcinoma patients admitted to our hospital from 2015 to 2023, as shown in Table 1, including age distribution, tumor size, FIGO stage, pathological type, degree of differentiation, vascular cancer thrombus, lymph node metastasis, HPV infection type, and reinfection status. From the table, we can see that the minimum age of the patient is 24 years, the maximum age is 89 years, and the average age is 51.34 years. The high-risk age group for cervical cancer is concentrated between 41 and 60 years. The pathological type of the patient is mainly squamous cell carcinoma. There are 343 cases of SCC, accounting for 85.8%, 52 cases of ADC, accounting for 13%, and 5 cases of other pathological types, accounting for 1.3%. Except for 143 patients who did not undergo standardized HPV examinations before surgery or were unable to track the results, in the HPV infection type statistics, there were 174 patients with a single HPV infection, 65 patients with two types of HPV infection, and 18 patients with three or more types of HPV infection. HPV multiple infections account for approximately one-third of HPV-infected patients, which also proves that HPV multiple infections are relatively common. Among 129 patients who could be traced back to postoperative follow-up results, 17 patients were reinfected with HPV, accounting for ~13.18%, and 112 patients were persistently negative, accounting for ~86.82%. The average follow-up time was 28 months.

**Table 1:** Clinical and pathological parameters of all subjects.

**Parameter**		***N***	**(%)**
Age (years)	≤ 30	13	3.3
	31–40	61	15.3
	41–50	105	26.3
	51–60	132	33.0
	61–70	76	19.0
	>70	13	3.3
FIGO stage	I	244	61.0
	II	90	22.5
	III	40	10.0
	IV	3	0.8
	Not reported	23	5.8
Tumor size	< 4 cm	186	74.70
	≥4 cm	63	25.30
	Not reported	151	-
Histological type	Squamous cell carcinoma (SCC)	343	85.8
	Adenocarcinoma (ADC)	52	13.0
	Other	5	1.3
	Yes	130	40.50
Vascular cancer thrombus	No	191	59.5
	Not reported	79	-
lymph node metastasis	Yes	53	16.25
	No	273	83.74
	Not reported	74	-
Infection type	Single subtype	174	67.7
	Two subtypes	65	25.29
	Three or more subtypes	18	7.00
	Not reported	143	-
Degree of differentiation	Poorly differentiated	38	16.24
	moderately differentiated	190	81.20
	Well differentiated	6	2.56
	Not reported	166	-
HPV reinfection	Yes	17	13.18
	No	112	86.82
	Not reported	271	-

### Correlation analysis between age and HPV infection types and distribution of common infection subtypes

We analyzed the distribution of HPV infection types in different age groups and found that the age group with a high incidence of HPV infection is between 30 and 70 years, with the age group of 41 to 60 years being more significant, which is basically consistent with the distribution of the high incidence age of cervical cancer. Among the multiple infections, most of the patients are younger than 40 years (≤ 30 years old: 44.4%, 31–40 years old: 38.8%) and older than 70 years (66.7%). We speculate that this may be related to factors such as sexual activity, fluctuations in hormone levels, decreased immunity, and an increased probability of causing latent viral infections. Among patients with multiple HPV infections, there are mainly 65 cases of double infection, 12 cases of triple infection, and fewer cases of four or more HPV infections. One patient is simultaneously infected with seven types of HPV. There is a significant difference in the distribution of HPV infection types among different age groups (*P* < 0.05) (Tables 2, 3).

**Table 2:** Distribution of HPV infection types in different age groups (N %).

**Infection type**	** ≤ 30**	**31–40**	**41–50**	**51–60**	**61–70**	**>70**	**Total**
Single subtype	5 (55.6)	30 (61.2)	50 (80.6)	54 (64.3)	33 (70.2)	2 (33.3)	174 (67.7)
Two subtypes	4 (44.4)	16 (32.7)	10 (16.1)	24 (28.6)	10 (21.3)	1 (16.7)	65 (25.3)
Three subtypes	0 (0.0)	2 (4.1)	2 (3.2)	4 (4.8)	1 (2.1)	3 (50)	12 (4.7)
Four subtypes	0 (0.0)	0 (0.0)	0 (0.0)	2 (2.4)	1 (2.1)	0 (0.0)	3 (1.2)
Five subtypes	0 (0.0)	1 (2.0)	0 (0.0)	0 (0.0)	1 (2.1)	0 (0.0)	2 (0.8)
Six subtypes	0 (0.0)	0 (0.0)	0 (0.0)	0 (0.0)	0(0.0)	0 (0.0)	0 (0.0)
Seven subtypes	0 (0.0)	0 (0.0)	0 (0.0)	0 (0.0)	1 (2.1)	0 (0.0)	1 (0.4)
Total	9 (100.0)	49 (100.0)	62 (100.0)	84 (100.0)	47 (100.0)	6 (100.0)	257 (100.0)

**Table 3:** Distribution of HPV infection types in different age groups (N %).

**Infection type**	** ≤ 30**	**31–40**	**41–50**	**51–60**	**61–70**	**>70**	**total**	**χ^2^**	***P***
Single subtype	5 (55.6)	30(61.2)	50(80.6)	54 (64.3)	33(70.2)	2(33.3)	174 (67.7)	26.5	0.003
Two subtypes	4 (44.4)	16(32.7)	10(16.1)	24 (28.6)	10 (21.3)	1(16.7)	65 (25.3)		
Multiple infections	0(0)	3(6.1)	2(3.2)	6(7.1)	4(8.5)	3(50)	18(7)		
Total	9 (100)	49(100)	62(100)	84(100)	47 (100)	6(100)	257 (100)		

We analyzed the distribution of HPV infection subtypes and found that the top five HPV subtypes with high detection rates in this study belonged to high-risk subtypes, namely, the HPV16, 18, 58, 33, and 52 subtypes (Table 4). Patients with multiple HPV infections are mainly infected with HPV16 mixed with other types of infection (referred to as “high-risk mixed type”), including mixed infections of high-risk and high-risk HPV and mixed infections of high-risk and low-risk HPV. According to the statistics on the number of types of HPV infections, the proportion of double infections is the highest. The top three HPV subtypes in dual infection include 16&18, 16&33, and 16&58 subtypes (Table 5). It is suggested that more attention should be paid to patients infected with the abovementioned HPV subtypes in our clinical studies.

**Table 4:** Distribution of HPV infection subtypes in different age groups (N).

**Infection subtype**	** ≤ 30**	**31–40**	**41–50**	**51–60**	**61–70**	**>70**	**Total**
16	8	35	43	66	33	5	190
18	3	11	8	9	8	0	39
58	1	3	2	7	9	1	23
33	0	5	2	10	2	1	20
52	0	3	3	6	2	1	15
31	0	3	2	3	1	1	10
39	1	2	3	0	0	1	7
53	0	1	0	2	3	1	7
81	0	0	2	0	4	0	6
51	0	0	1	3	1	0	5
59	0	2	1	2	0	0	5
42	0	0	1	2	1	0	4
45	0	1	0	3	0	0	4
56	0	0	1	3	0	0	4
66	0	0	1	0	2	1	4
11	0	2	0	0	1	0	3
35	0	0	1	1	1	0	3
68	0	1	1	0	1	0	3
82	0	1	1	0	0	0	2
CP8034	0	0	0	2	0	0	2
13	0	0	1	0	0	0	1
26	0	0	0	0	0	1	1
43	0	0	0	1	0	0	1
44	0	0	0	0	1	0	1
67	0	0	0	1	0	0	1
73	0	1	0	0	0	0	1

**Table 5:** Distribution of HPV multiple genotypes.

**HPV genotypes**	**Cases, *n* (%)**
16‘18	12 (14.5)
16‘33	9 (10.8)
16‘58	9 (10.8)
16‘52	5 (6.0)
16‘39	3 (3.6)
16‘53	3 (3.6)
16‘31	2 (2.4)
18‘31	2 (2.4)
16‘81	2 (2.4)
16‘51	2 (2.4)
16‘CP8034	2 (2.4)
16‘33‘52	2 (2.4)
16‘18‘45	2 (2.4)
Others	29 (34.9)

### Lymph node metastasis and vascular cancer thrombus

We used SPSS software version 25.0 for statistical analysis of lymph node metastasis and vascular cancer thrombi. A heterogeneity comparison between the groups was conducted using the chi-square test, and Fisher's exact test was used when a single sample size was < 4. For the remaining analyses, a *p*-value of < 0.05 was considered statistically significant. Table 6 shows the relationship between lymph node metastasis and vascular tumor thrombus with age, HPV infection type, tumor size, and tumor stage. It is found that there is no significant difference between age and HPV infection type groups, while there is a significant difference between tumor size and stage groups. From Table 7, we can see that patients with vascular cancer thrombi are more prone to lymph node metastasis, and there is a significant difference between the two groups.

**Table 6:** Chi-square test.

**Parameter**		**Lymph node metastasis**			**Vascular cancer thrombus**		
		**N (%)**	**χ** ^2^	***p***	**N (%)**	**χ** ^2^	***p***
Age	30	3(33.3)	8.258	0.143	2(40.0)	1.783	0.878
	31–40	6(14.3)			15(34.1)		
	41–50	17(19.3)			34(40.0)		
	51–60	22(19.3)			48(42.1)		
	61–70	4(6.3)			25(40.3)		
	>70	1(10.0)			6(54.5)		
Infection type	HPV single	17(11.1)	0.056	0.810	59(37.8)	0.037	0.847
	HPV multiple	7(12.3)			22(39.3)		
Tumor size	< 4cm	29(16.6)	12.137	0.002	82(48.5)	23.667	0.000
	≥4cm	17(29.8)			30(53.6)		
FIGO stage	I	11(5.5)	144.410	0.000	64(31.4)	25.502	0.000
	II	13(15.3)			46(56.1)		
	III and IV	29(90.6)			19(70.4)		

**Table 7:** Chi-square test.

	**Lymph node metastasis**			**χ^2^**	***p***
Vascular cancer thrombus		Yes	No	28.862	0.000
	Yes	36	93		
	No	9	159		

### Correlation analysis between multiple HPV infections and cervical cancer factors

We conducted correlation analysis based on different groups of multiple infections and found that there was no significant relationship between HPV multiple infections and cervical cancer stage, lesion size, pathological tissue type, tissue differentiation degree, vascular cancer thrombus, and lymph node metastasis. There was no significant difference in the results between the groups (Tables 8, 9).

**Table 8:** Analysis of clinical and pathological parameters of multiple HPV infections and cervical cancer.

**Correlations**	**0–1 grouping**	**Single double multiple grouping**	**Expanded subdivision**
Age	Pearson Correlation	0.01	0.067	0.089
	Sig. (2-tailed)	0.878	0.284	0.157
	N	257	257	257
Tumor size	Pearson Correlation	−0.038	−0.019	−0.022
	Sig. (2-tailed)	0.555	0.776	0.731
	N	239	239	239
FIGO stage	Pearson Correlation	0.004	−0.027	−0.033
	Sig. (2-tailed)	0.948	0.681	0.606
	N	240	240	240
Vascular cancer thrombus	Pearson Correlation	0.013	0.031	−0.016
	Sig. (2-tailed)	0.85	0.664	0.825
	N	204	204	204
Lymph node metastasis	Pearson Correlation	0.008	−0.011	−0.026
	Sig. (2-tailed)	0.913	0.876	0.718
	N	202	202	202
Degree of differentiation	Pearson Correlation	−0.010	−0.012	0.020
	Sig. (2-tailed)	0.908	0.886	0.809
	N	144	136	149

**Table 9:** Analysis of multiple HPV infections and pathological types.

		**ADC**	**SCC**	**Total**	**χ2**	***p***
0–1 grouping	Single subtype	26	145	171	1.493	0.222
	Non-single infection	8	75	83		
Single double multiple grouping	Single subtype	26	145	171	3.335	0.189
	Double subtypes	8	57	65		
	Multiple subtypes	0	18	18		
Expand Subdivision	Single subtype	26	145	171	3.335	0.649
	Double subtypes	8	57	65		
	Three subtypes	0	12	12		
	Four subtypes	0	3	3		
	Five subtypes	0	2	2		
	Six subtypes	0	0	0		
	Seven subtypes	0	1	1		
	Total	34	220	254		

## Discussion

### Multiple HPV infections and cervical carcinoma

Cervical carcinoma is a usual malignancy in the female reproductive system. It is one of the malignant tumors with high morbidity and mortality among women in developing countries, threatening the health of many women. According to the statistics, ~48,000 women die from cervical cancer every year (Chen et al., 2016; Bray et al., 2018). At present, it has been determined that the sustained infection of human papillomavirus is a major influencing factor for the occurrence of cervical carcinoma, and we can determine the type and subtype of HPV infection through cervical screening. There are over 200 subtypes of HPV found currently, and 40 subtypes closely related to the reproductive tract (Chunyou and Wang, 2019). According to their risk of causing cervical carcinoma, HPV can be divided into high-risk and low-risk types. The former includes 16, 18, 31, 33, 35, 39, 45, 51, 52, 56, 58, 59, and 68 (Vaccine Immunization Branch of Chinese Preventive Medicine Association, 2019). Persistent infection of high-risk HPV is closely related to cervical carcinoma, while low-risk HPV is more common in benign lesions such as genital warts. According to the reports, persistent infection of high-risk HPV in the cervix can lead to precancerous lesions and can further develop into invasive cancer, accounting for 99.7% of cervical carcinoma cases infected with high-risk HPV (Andersson et al., 2005).

Multiple infections refer to the simultaneous infection of two or more HPV subtypes, which may be secondary, tertiary, or even more. The inconsistent pathogenicity of the different subtypes of HPV indicates a different risk of cervical lesions. Therefore, many scholars are paying attention to the correlation between multiple HPV infections and cervical carcinoma (Salazar et al., 2015). A recent overseas project showed that 622 cases are multiple infections in 1,216 patients with HPV infection, accounting for almost half of the total (Kim et al., 2021). Our research results also indicate that multiple HPV infections in the cervix are common. However, there is no consensus on whether there is a cooperation between multiple HPV infections and whether it has a promoting effect on the development of cervical carcinoma at various stages. Some scholars believe that multiple HPV infections are related to the progression of cervical lesions (Chowell et al., 2012; Yokomichi et al., 2018; Homaira et al., 2019; Tramuto et al., 2019; Ghimire and Moon-Grady, 2020), but others highlight that there is no correlation between multiple HPV infections and cervical lesions (Xu et al., 2012; Tagarro et al., 2019).

### HPV pathogenesis and synergistic effects between multiple infections

HPV infects the cervical epithelial cells through micro damage, which enters and spreads to the basal layer of the cervical epithelium. HPV is released from the capsid and enters the nucleus, becoming a free gene. HPV exists in three forms after infecting cervical epithelial cells, including free type, integrated type, and mixed type. When HPV is in an integrated state, it can exist in the body for a long period of time, making it less likely to be detected by the host immune system and becoming a “persistent infection”, which is more common in high-grade intraepithelial neoplasia and cervical carcinoma. Some studies have shown that as the degree of cervical lesions worsens, the proportion of integrated types gradually increases, while the proportion of free types gradually decreases. Especially in cervical cancer, multiple integrated types account for the majority. In cervicitis and CIN, single integration is predominant (Huang et al., 2017; Groves and Coleman, 2018; Xia et al., 2020).

Important genital HPV genotypes belong to α Papillomavirus genus, including α-9, α-7, α-6, and α-5 four categories. α-9 included the high-risk HPV subtypes 16, 31, 33, 35, and 52, and α-7 included the high-risk HPV subtypes 18, 39, 45, 59, and 68. Some studies have shown that multiple infections of genotype α-9 increase the risk of cervical carcinoma by 5.3 times, and multiple infections of genotype α-7 increase the risk of cervical carcinoma by 2.5 times, which also explain that the same type of HPV genotype may have a synergistic effect on co-infection to induce cervical carcinoma (Saslow et al., 2012). Iacobone et al. Iacobone et al. (2019) found that there is a synergistic effect between specific genotypes among different subtypes of HPV, with the association between HPV16 and 53 being more pronounced. However, Vinokurova et al. (Vinokurova et al., 2008) found that HPV16, 18, and 45 have more integration potential compared to HPV31 and 33, which makes them more likely to cause high-grade lesions to progress to cervical carcinoma. HPV16 is present in most multiple infections. HPV16/18 and HPV16/31 are the most common combination of mixed infections (Aleksioska-Papestiev et al., 2018). The research by de Freitas et al. (de Freitas et al., 2015) shows that the most common integrated subtypes in northwestern China are HPV16, 58, and 33. The results of our study show that the incidence of multiple HPV infections is 32.29%, in which HPV16 mixed with other types of infection is the main type, basically consistent with the above research results. Due to the limited sample capacity of this study, we need to conduct large-scale multicenter epidemiological surveys in the future to gain a more comprehensive knowledge of the correlation between HPV multiple infections and cervical lesions and cancer.

### Multiple HPV infections and age

An abundance of research studies have shown that HPV infection is age-related, and multiple infections show different characteristics at different ages, with a central tendency trend of age (Guo et al., 2007; Saslow et al., 2012). Anna et al. (Iacobone et al., 2019) showed a negative correlation between age and the number of HPV infection types, proving that multiple infections typically happen to sexually active young women. However, some literature studies show that the incidence of HPV multiple infections is higher in elderly patients with cervical carcinoma, which may be related to the decrease in the immune system and significant changes in hormone levels (Saslow et al., 2012). The results of our study show that most of the patients with multiple HPV infections are younger than 40 years (≤ 30 years old: 44.4%, 31–40 years old: 38.8%) and older than 70 years (66.7%). We speculate that this may be related to factors such as sexual activity, fluctuations in hormone levels, decreased immunity, and an increased probability of causing latent viral infections. Our results are consistent with previous reports. In our study, the HPV16, 18, 58, and 33 subtypes were the main subtypes among patients with multiple infections, with HPV16 being the most predominant. Biryukov et al. (Biryukov and Meyers, 2018) found that HPV16 is more likely to adhere to the surface of cervical epithelial cells and enter them, which may be the cause for the higher incidence of infection, compared to other types. Therefore, we believe that multiple infections are mainly high-risk mixed infections, and for mixed infections of the HPV16, 18, 58, and 33 subtypes, we should pay more attention to the clinical treatment.

### Multiple HPV infections and prognosis

Joo et al. used *in situ* hybridization and polymerase chain reaction (PCR) to detect HPV integration status and prognosis in 204 patients undergoing chemotherapy for cervical carcinoma. The authors found that HPV integration is a biomarker that predicts disease-free survival after cervical carcinoma (Joo et al., 2017). From this, we can see that the integrated state of HPV may be regarded as one of the markers of disease progression. In addition, there are also reports that HPV integration occurs at the initial stage of cervical lesions, and the mixed type is more likely to predict disease progression than the fully integrated type. Some scholars have found that squamous cell carcinoma has a higher HPV integration rate compared to adenocarcinoma. HPV integration provides a selective growth superiority in infected cells, which is related to the failure of therapy and shortened disease-free survival (Flores, 2006; Guo et al., 2007; Joo et al., 2017). However, the association between the integration status of HPV and the progression of cervical lesions still needs further research. DE BROT et al. (De Brot et al., 2017) suggest that there are multiple HPV infections in cervical lesions; however, there is still a lack of clear correlation between them and cervical histology. The results of this study suggest that there is no significant difference between single and multiple HPV infections in terms of pathological classification, tissue differentiation, FIGO staging, tumor size, vascular cancer thrombi, and lymph node metastasis. We believe that the prognosis of cervical cancer is influenced by multiple factors such as diagnostic timing, treatment methods, tumor characteristics, and HPV integration and is the result of a combination of multiple factors.

## Conclusion

In summary, multiple types of HPV infection in the cervix are common. However, there is currently no consensus at home or abroad on the occurrence, development, and impact of multiple HPV subtypes of infection on cervical lesions and cervical carcinoma. This study found that multiple infections, mainly HPV16, are closely interrelated to cervical carcinoma. For the HPV16, 18, 58, 33, and 52 subtypes of infection, especially for patients younger than 40 years and older than 70 years who have multiple infections with the HPV16, 18, 58, and 33 subtypes, priority should be given to close follow-up, prevention, and active treatment. The relation between multiple HPV infections and the evolvement and prognosis of cervical carcinoma requires a large sample study from multiple centers, which could better guide cancer prevention and treatment.

## Data availability statement

The original contributions presented in the study are included in the article/supplementary material, further inquiries can be directed to the corresponding authors.

## Ethics statement

The studies involving human participants were reviewed and approved by the Ethics Committee of the Second Affiliated Hospital of Dalian Medical University. The patients/participants provided their written informed consent to participate in this study.

## Author contributions

JL and SH processed the case and drafted the manuscript. JN and YL assisted in collecting case data and literature, and conducted all literature and article work. JW made many constructive suggestions during the revision and production process. XW contributes to the statistical analysis of case data. All authors contributed to the article and approved the submitted version.
